# Anthropometric assessment of obesity in robotic‐assisted laparoscopic prostatectomy: A systematic review

**DOI:** 10.1002/bco2.70206

**Published:** 2026-05-11

**Authors:** Ashley Lee, Elizabeth Crostella, Ann Grand, Matthew Brown

**Affiliations:** ^1^ University of Western Australia Perth Australia; ^2^ East Metropolitan Health Service Perth Australia; ^3^ University of the West of England Bristol UK

**Keywords:** abdominal adiposity, anthropometric methods, obesity, RALP, robotic prostatectomy

## Abstract

**Introduction:**

Obesity rates are rising among prostate cancer patients undergoing robotic‐assisted laparoscopic prostatectomy (RALP). This review evaluates the anthropometric tools used to study this group and their correlation with surgical outcomes. Our objective is to identify which obesity metrics have been measured against clinically relevant surgical outcomes in patients undergoing RALP to enable more accurate risk stratification and improved patient counselling.

**Methods:**

Five databases (Ovid, Medline, Web of Science, Cochrane, Scopus) were systematically searched in March 2025 for studies examining obesity metrics and RALP outcomes. Inclusion criteria: English‐language studies from the last 15 years with ≥15 patients undergoing transperitoneal RALP. Two independent reviewers screened articles and assessed bias using the Newcastle–Ottawa Scale. Data on anthropometric tools, patient demographics, obesity definitions and outcomes were extracted and analysed descriptively.

**Results:**

Search strategy yielded 15 papers in total demonstrating that body mass index (BMI) is the only anthropometric tool used in surgical research to assess obese patients for relevant outcomes from RALP. Outcomes observed were categorised as functional, oncological and perioperative. Most studies examined a combination of all three.

**Conclusions:**

The data consistently utilises BMI to measure obesity in RALP patients, with numerous surgical outcomes explored in the literature. However, there is a paucity of studies examining alternative obesity metrics. Given the abdomino‐pelvic site of prostatectomy, metrics that more accurately assess body fat distribution in this anatomical area may be more appropriate for research and clinical practice.

## 1 INTRODUCTION

Surgical practice must adapt to the rising incidence of obesity by relying on robust evidence. Research should accurately assess the safety and efficacy of operating on obese patients. Men aged 60–79 are most likely to be diagnosed with prostate cancer,^1^ with over 80% of men in this age group classified as obese.^2^ For surgical candidates, robotic‐assisted laparoscopic prostatectomy (RALP) is considered the superior treatment for organ‐confined prostate cancer.^3^ Obesity complicates RALP in several ways. Excess adipose tissue increases the time to dissect the Retzius space and the difficulty of dissecting structures during the procedure, incurring higher volume blood loss and limited intra‐abdominal working area complicates the urethrovesical anastomosis.^4^ A narrowed and deeper true pelvis and increased distance from skin to working area result in less‐than‐optimal visualisation,^5^, ^6^ which may be linked to higher rate of positive surgical margins.^7^ Nerve sparing is classified as ‘very difficult’ in morbidly obese patients.^8^ Due to these challenges, it is recommended that only experienced surgeons perform the operation on obese patients.^6^, ^9^

Despite these technical challenges, evidence linking obesity to surgical complications in RALP remains inconsistent. Early research indicated that body mass index (BMI) was not a predictor of complications in RALP.^10^ However, Kim et al.^11^ found higher rates of biochemical recurrence post prostatectomy in overweight or obese men, while Khaira et al.^12^ and Mikhail et al.^13^ have statistically significant opposing conclusions on operating time. These heterogeneous findings raise questions about the validity of using BMI to assess obesity in abdominopelvic surgery outcomes. Various tools and metrics are used to measure obesity in clinical practice and research, each with unique benefits and limitations. Visceral fat area (VFA), measured with computed tomography or magnetic resonance imaging, is the gold standard for measuring abdominal adiposity.^14^ Waist to hip ratio (WHR) and waist circumference (WC) manually calculate body fat distribution around the visceral area, with high WC being a reliable surrogate measurement of VFA.^15^ Dual‐energy x‐ray absorptiometry estimates body composition and is a valid and reliable way of measuring abdominal obesity.^17^ BMI, measured as weight (kg)/height(m^2^),^20^ is the most used obesity metric in healthcare but does not describe body fat distribution. The WHO classifies weight categories in kg/m^2^ as: underweight <18.5, normal weight 18.5–24.9, overweight 25–29.9 and obesity into class I: 30–34.99, class II: 35–39.99 and class III: >40.^21^ Given the variety of available anthropometric tools, researchers should select the metric that most appropriately addresses their specific research question and anatomical site of interest.

Our objective is to identify which obesity metrics and categories of surgical outcomes are most appropriate for robust investigation of obesity's role in RALP. The primary research question for this systematic review is: For patients undergoing RALP, which obesity metrics have been measured against clinically relevant surgical outcomes? The secondary research question is: In relation to obesity, which clinically relevant surgical outcomes have been explored in the literature? This systematic review evaluates obesity metrics and their association with RALP outcomes. Findings will enable more accurate risk stratification and improved patient counselling in prostate cancer surgery.

## 2 MATERIALS AND METHODS

A systematic review was conducted to thoroughly investigate the available literature for all anthropometric tools and clinical outcomes that have analysed this patient population. This systematic review was conducted in accordance with the PRISMA reporting guidelines and registered on PROSPERO number: CRD420251060181.

We have included studies examining robotic prostatectomy and obesity‐related surgical outcomes only, published within the last 15 years, available in full text, in or translatable to English, including case series, any methodology of study which sought to answer the research question directly as a primary or secondary objective and standardised to a transperitoneal approach. Excluding case studies and case–control studies, any other surgical approach or compared approaches, costing analyses, non‐published findings, conference proceedings and series that reviewed less than 15 patients.

The following search terms, key words and MeSH terms were used: obesity, obese* OR obesity measurement tools, obesity metrics, anthropometrics, overweight, Visceral fat adiposity, adipos*, visceral adiposity, abdominal circumference, skin fold thickness, body fat percentage, waist to hip ratio, waist circumference, BMI, body mass index, dual‐energy x‐ray absorptiometry (DEXA), bipolar bioelectrical impedance analysis (BIA) AND (robotic assisted laparoscopic prostatectomy) OR robotic prostatectomy, robotic laparoscopic radical prostatectomy, RLRP, RALP AND clinically relevant surgical outcomes OR blood loss, bleeding, operative time, blood transfusion, blood products, complications, ‘intraoperative complications’, ‘postoperative complications’, margin status, positive margin, biochemical recurrence, length of stay, open conversion rates, time with catheter and estimated blood loss. The literature search explored the following databases: Ovid, Medline, Web of Science, Cochrane library, Scopus and a bibliographic review from systematic review(s) on the topic.

**FIGURE 1:**
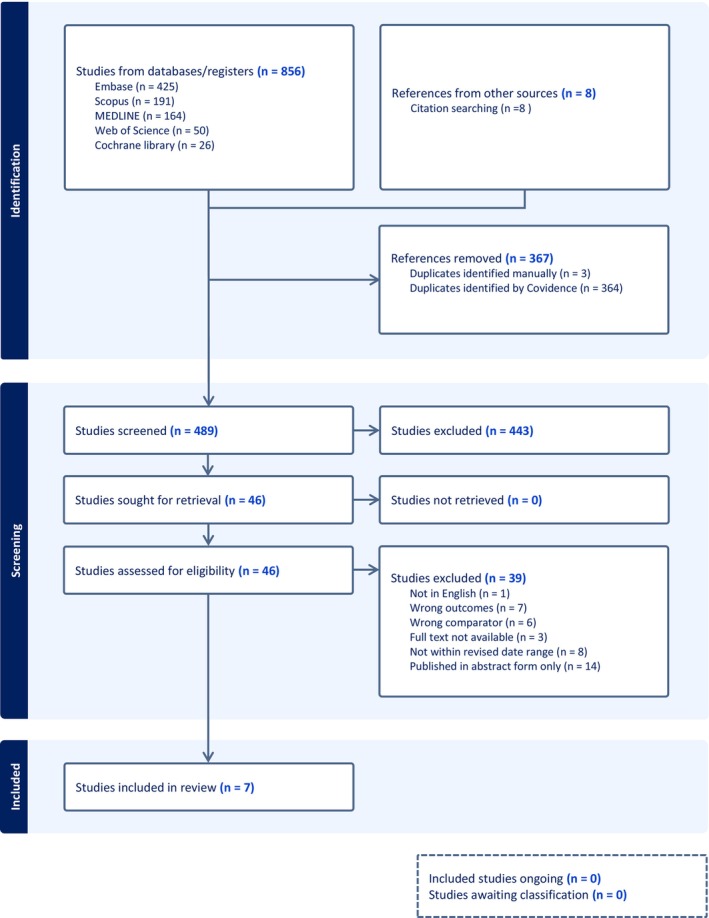
PRISMA flow diagram.

The search strategy was conducted on 27 March 2025, with the detailed search strategy seen in Table S1. A PRISMA flow chart seen in Figure 1 has been used to demonstrate the identification and screening process for the studies included. A second reviewer was involved to review the articles for screening and selection for extraction, as well as assess for potential confounders and biases using the Newcastle–Ottawa Scale. Inconsistencies between reviewers were determined by the primary author. Statistical analysis is descriptive, excluding meta‐analysis as out of scope for the research question.

The results were tabulated and analysed comparing the anthropometric tool used. Demographics of the patients, details of study design, definition of obesity and the clinically relevant surgical and oncological outcomes were collected for analysis. Common themes with high statistical significance are explored as well as study characteristics and funding sources.

## 3 RESULTS

Eight hundred fifty‐six articles were identified, and 489 were retrieved for title and abstract screening; 46 studies were deemed suitable, and 39 were excluded. Reasons for exclusion can be seen in the PRISMA diagram in Figure 1. A total of seven studies were yielded from the search strategy. The following articles were found through bibliographic cross‐referencing,^5^, ^8^, ^9^, ^22^, ^23^, ^24^, ^25^, ^26^ resulting in 15 studies for inclusion in the analysis.

The studies were relatively consistent when describing the demographics of their patient population. Age, prostate‐specific antigen, clinical and pathological staging, prostate size and comorbidity were commonly collected demographics. D'Amico risk categorisation, ethnicity, history of abdominal or prostate surgery and income level were rarely used.

The Newcastle–Ottawa bias assessment is reported in Table S2 and suggests an overall low risk of bias with occasional risk, demonstrating overall good quality studies with adequate selection, comparability and outcome assessment. Most studies reported no conflict of interest, two studies omitted any formal statement regarding competing interests, and the remainder formally declared none. Most studies had no funding source declared. Three studies were funded by institutional grants or sponsored by state health research funding. However, one study^9^ received a clinical research grant from Intuitive Surgical, the manufacturer of robotic surgical systems. While the authors declared no conflicts of interest and the study design appeared methodologically sound, the presence of industry sponsorship warrants cautious interpretation.

**FIGURE 2:**
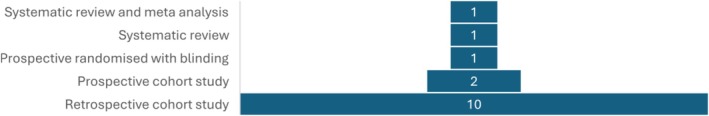
Hierarchy of evidence identified in systematic review.

Figure 2 illustrates the imbalance in the hierarchy of evidence on this topic. Many studies are retrospective observational cohort studies, with only one systematic review that includes a meta‐analysis. The publication dates range from the inclusion date of 2010 to the most recent in 2025.

All 15 studies used BMI as the sole obesity metric in the RALP cohort, with no other metrics examined. The authors did not provide specific rationale for choosing BMI. Obesity was consistently defined as >30 kg/m^2^. Some studies dichotomised data into obese or not obese using 30 as the cut‐off, while others categorised data with greater granularity. Heterogeneity of obesity assessment can be seen in Table 1.

**TABLE 1:** Demographics.

Author and year	Study design	BMI categories (kg/m^2^)	Number of patients	Median age in years (IQR)	Mean initial PSA (ng/mL)	Prostate size (gm)
Jaber 2025	Retrospective cohort study (propensity score matched)	Normal (18.5–24.9)	183	61 (54–67)	5.4 (4.1–8.2)	48 (41–61)
		Morbidly obese (>40)	183	60 (55–65)	5.7 (4.5–8.9)	54 (44–64)
Wang 2024	Systematic review and meta‐analysis	Non‐obese	55 266	60.8	—	49.72
		Obese	8434	57.9	—	53.78
Farzat 2023	Prospective cohort study	Normal (<30)	336	68 (61.25–72)	7.7 (8–24)	41 (31–56)
		Obese (≥30)	164	68 (62–72)	9 (5.6–14.9)	47 (31–56)
Mourão 2022	Retrospective cohort study	Normal (18.5–24.9)	295	62 (57–68)	5.4 (4.0–7.84)	39 (30–48)
		Overweight (25–29.9)	535	61 (56–67)	5.47 (4.01–7.75)	41 (34–51)
		Obese (≥30)	247	61 (57–66)	5.53 (4.01‐8.4)	42 (34–54)
Sarychev 2022	Prospective cohort study (propensity score matched)	Normal (18.5–24.9)	1701	64 (59–69)	7.6 (5.6‐11)	—
		Obese (≥30)	1701	64 (59–69)	7.4 (5.4–11)	—
Goßler 2020	Prospective, randomised, single‐blind series	Non‐obese (<30)	181	65 (60–70)	8 (6–12.8)	49 (40–66)
		Obese (≥30)	51	67 (62–70)	9 (6.2–14.4)	55 (41–71)
Han 2020	Retrospective review of database	Non‐obese	48 725	61.74	—	—
		Obesity class I–II	3572	61.21	—	—
		Morbid obesity	1004	59.99	—	—
Garg 2017	Retrospective cohort study	Normal (18.5–24.9)	101	59.1	5.38 (4.19–7.17)	30 (21.49–42.68)
		Overweight (25–29.9)	300	59.6	4.96 (3.80–7.09)	31.94 (26.05–42.18)
		Obese (≥30)	290	58.7	5.19 (4.17–7.03)	35.82 (28.40–47.98)
Xu 2015	Systematic review	Non‐obese	4801	60.6	—	49.05
		Obese	1821	59.6	—	52.37
Abdul 2014	Retrospective cohort study from database	Not morbidly obese (<40)	44	57.9	6.0	50.9
		Morbidly obese (≥40)	44	58.7	5.9	53.6
Gu 2014	Prospective cohort study	Normal (<25)	36	61.8 (49–72)	6.1	41.2
		Overweight (25–29.9)	115	63.1 (45.3–78.1)	6.7	42.6
		Obese (≥30)	67	62.7 (47–75.1)	5.5	43.7
Zilberman 2012	Retrospective cohort study	Normal (<25)	102	60.1 (55.1–64.5)	5.3 (3.9–7.1)	
		Overweight (25–29.9)	301			—
		Obese (≥30)	174			
Yates 2011	Retrospective cohort study	Morbidly obese (≥40)	15	—	5.78	48.5
Zilberman 2010	Retrospective cohort study	<25	100	61 (56–67)	5.2 (3.9–6.8)	44 (36–58)
		25–29.9	286	60 (55–64)	5.4 (4.0–7.1)	44 (36–56)
		30–34.9	135	60 (55–64)	4.8 (3.8–6.9)	47 (38–59)
		≥35	34	59 (54–63)	5.5 (3.7–7.7)	53 (41–64)
Moskovic 2010	Retrospective cohort study (prospective database)	Normal (<25)	270	61	5.0	48
		Overweight (25–29.9)	600	60	5.0	53
		Obese (≥30)	242	59	5.2	56

We identified the clinically relevant surgical outcomes of interest to researchers. They are categorised into peri‐operative, functional and oncological outcomes. Many of the studies investigate more than one category.

Details and frequency of outcomes reviewed can be seen in Table 2. The most popular outcomes were blood loss, operative time, continence and positive surgical margins. Table 3 displays the primary endpoints reviewed by the respective studies, demonstrating a preponderance towards assessing a heterogeneous mix of varied peri‐operative outcomes.

**TABLE 2:** Frequency of literature results for BMI and RALP outcomes.

Outcome	No. of studies that reviewed the outcome	Referenced studies
Perioperative		
Pulmonary complications	1 (7%)	[5]
Bladder neck contracture	1 (7%)	[24]
Aborted procedure	1 (7%)	[25]
DVT	1 (7%)	[24]
Readmission	1 (7%)	[22]
Anastomotic leak	2 (14%)	[22, 24]
Lymphocele	2 (14%)	[4, 22 22]
Other intra‐/peri‐operative complications	3 (20%)	[5, 24, 26]
Open conversion	3 (20%)	[8, 24, 25]
Catheter days	3 (20%)	[4, 8, 22]
Transfusion	5 (34%)	[8, 9, 22, 24, 25]
Length of stay	8 (54%)	[4, 5, 8, 21, 22, 23, 26, 27]
Clavien–Dindo classification	9 (60%)	[4, 8, 9, 19, 20, 21, 22, 23, 25]
Estimated blood loss	11 (74%)	[4, 8, 9, 21, 22, 23, 25, 26, 27, 28]
Operative time	11 (74%)	[4, 8, 9, 19, 21, 22, 23, 24, 25, 26, 27]
Functional		
Urinary symptoms	1 (7%)	[26]
Quality of life	2 (14%)	[4, 24]
Potency/erectile function	9 (60%)	[4, 8, 9, 21, 23–26, 28]
Continence	11 (74%)	[4, 8, 9, 19, 20, 21, 23, 24, 25, 26, 28]
Oncological		
Metastatic progression	1 (7%)	[9]
Biochemical recurrence	4 (27%)	[4, 9, 24, 26]
Pre‐ to post‐operative tumour grading	7 (47%)	[4, 8, 9, 24, 25, 26, 28]
Positive surgical margin	12 (80%)	[4, 8, 9, 19, 21, 22, 23, 24, 25, 26, 27, 29]

**TABLE 3:** Primary endpoints for all included studies.

	Primary endpoint						
	Perioperative			Functional		Oncological	
	Complications (Clavien–Dindo)	Other outcomes	Readmission	Continence	Potency	Biochemical recurrence	Positive surgical margin
Jaber et al. 2025	✓	—	—	—	—	—	—
Wang et al. 2024	—	✓	—	—	—	—	—
Farzat et al. 2023	✓	—	✓	—	—	—	—
Mourão et al. 2022	—	—	—	✓	—	—	—
Sarychev et al. 2022	—	—	—	—	—	✓	—
Goßler et al. 2020	—	—	—	—	—	—	✓
Han et al. 2020	—	✓	—	—	—	—	—
Garg et al. 2017	—	—	—	✓	✓	—	—
Xu et al. 2015	—	✓	—	—	—	—	—
Abdul et al. 2014	—	✓	—	—	—	—	✓
Gu et al. 2014	—	✓	—	—	—	—	—
Zilberman et al. 2012	—	—	—	—	—	—	✓
Yates et al. 2011	—	✓	—	—	—	—	—
Zilberman et al. 2010	—	✓	—	—	—	—	—
Moskovic et al. 2010	—	✓	—	✓	✓	✓	✓

## 4 DISCUSSION

This systematic review reveals that BMI is the predominant anthropometric tool used in surgical research to assess outcomes in RALP patients. This preference may stem from the ease of obtaining height and weight measurements and the routine use of BMI in perioperative assessments by other disciplines such as anaesthetics and nursing. BMI is a ubiquitous marker for obesity in healthcare research, facilitating cross‐referencing and replication of findings. Given the relatively recent adoption of robotic surgery, researchers may have opted for a widely recognised and reliable metric to avoid complicating the data.

The definition and analysis of obesity varied across studies. Some included morbid obesity, others dichotomised data at BMI 30, and some reported across all BMI categories. This variability should be considered when comparing outcomes. For instance, including overweight individuals in the ‘non‐obese’ category may obscure the clinical outcomes specific to this group. Four studies directly investigated morbid obesity^4^, ^5^, ^8^, ^19^ but different outcome measures make meta‐analysis of their data difficult.

The literature highlights the challenges of operating on an obese abdomen.^9^ Studies using BMI as the sole measure of obesity failed to account for central adiposity. Abdominal and visceral fat obscure anatomical structures, complicating prostate excision compared to normal‐weight patients.^25^ Additionally, the restricted operative field in obese abdomens reduces the manoeuvrability of the robotic arm during surgery.^26^

A recent systematic review assessed obese patients undergoing RALP using BMI as the metric, revealing worse clinically significant outcomes in obese cohorts.^22^ The data in the studies could not have determined if central adiposity contributed to these outcomes. Other surgical specialties have sought more precise measurements of obesity relevant to the abdomino‐pelvic operative site.

Kuritzkes et al^33^ reviewed VFA and determined it was significantly associated with higher morbidity and complications in colonic resection. VFA was associated with 25% increased morbidity compared to BMI in colectomy patients.^34^ In another colectomy series Zhai et al^35^ found that higher VFA was associated with an increased incidence of overall postoperative complications by 32% compared to BMI. Specific complications such as anastomotic leakage, surgical site infection, and overall postoperative complications were higher in the obese VFA group compared to obese BMI.^36^ Additionally to VFA, WC and WRH were deemed superior to BMI in predicting morbidity and mortality in colorectal surgery.^37^ Bachmann et al^38^ recommend that VFA or WHR measurements should be routinely employed in clinical practice.

The RALP literature relies on BMI as the sole measure of obesity, which may be inadequate for predicting surgical outcomes, especially in cases of central abdominal obesity. This narrow approach contrasts with the diverse measurement tools used in colorectal series. More precise data on body fat distribution could better inform surgeons and patients about their specific risks associated with the operation. Additionally, perioperative, functional, and oncological risks can be mitigated through alternative treatments, such as radiation therapy, which may offer safer outcomes for obese patients while maintaining equivalent oncological results.^39^

Operative time and estimated blood loss were the most reviewed perioperative outcomes. 10 of the 15 studies in the systematic review showed a statistically significant increase in operative time, though this did not always correlate with perioperative complications. From a health economics perspective, longer operative times increase costs and may reduce the number of cases handled daily, affecting the oncological surgical waitlist. Obese men often have larger prostates, complicating the surgery. Specifically, the vesicourethral anastomosis time is longer and more technically challenging.^12^ Future research should stratify findings by prostate size, as this was not extensively explored in the current series. Similarly, seven out of 15 papers reported statistically significant higher blood loss in the obese groups, but this did not translate to higher rates of transfusion or differences in haemoglobin drop.^24^, ^27^

Despite an overall low risk of bias calculated in the Newcastle‐Ottawa assessment, there are some notable confounders in the body of literature. This review favours observational retrospective cohort studies. The lack of randomisation introduces bias through patient selection for surgery and the absence of a matched control group, making comparisons unreliable. Surgeons may delay surgery for obese patients, leading to later surgery dates compared to normal‐weight patients, resulting in more advanced disease progression. Clinician‐centred selection bias may also influence recommendation for surgical or alternative treatment strategies for obese prostate cancer patients. While this bias is a limitation, it also strengthens the research by reflecting real‐world conditions, thus enhancing the generalisability of the conclusions.^8^

Patient comorbidity is a significant confounding factor in the data sets, as preoperative chronic diseases can lead to poorer surgical outcomes independent of obesity.^40^ Five studies, including the systematic review by Wang et al^25^ of 36 700 patients in 16 papers, did not address comorbidity status, likely due to heterogeneity in reporting. Verified tools such as the Charlson Comorbidity Index, ASA score, and Elix Hauser Comorbidity Index were used by some studies to stratify patients into a single index score. In contrast, three studies included individual diseases such as diabetes, hypertension, and coronary artery disease as independent variables. Some efforts were made to address the confounding factors when obtaining research data from observational cohort studies. To isolate the effect of obesity five studies^4^, ^8^, ^9^, ^23^, ^24^ used propensity score matching algorithms to ensure like for like comparisons of the groups comorbidities to ensure that outcomes can be attributable to the obesity alone.

Follow‐up duration varied, with the longest being a median of 24.8 months.^27^ Many studies reported limitations in follow‐up beyond 12 months due to standard clinical practice of discharging patients to then general practitioners care at that point. This limited follow‐up highlights the constraints of retrospective research from medical records.

Positive surgical margins are influenced by multiple factors such as prostate size,^26^ surgeon experience,^41^ clinical stage of the disease and obesity obscuring anatomical landmarks such as the bladder neck.^29^ Most studies reporting positive margins require further analysis including discussion as to the role of these contributing factors. Additionally, there was minimal follow‐up on obese patients with positive margins to assess progression to clinically significant biochemical recurrence compared to non‐obese patients.

Surgical experience is a significant confounder in this data set, often overlooked in analysis and discussions. Inexperienced surgeons have longer operative times during their learning curve,^39^ and positive margin rates decrease with experience.^41^ Studies suggest that experienced surgeons can achieve favourable outcomes in RALP for obese patients.^8^ There is limited analysis on the impact of the learning curve and surgeon experience found in this review.

Limitations of this review include that a large body of research specifically looking at positive surgical margins and obesity was identified through the search strategy. They were decidedly not included due to being a separate mature body of research which the search strategy was not specifically directed to find. Another limitation is that this study did not aim to look at the internal validity of the research papers examined, and the significance of the findings was not explored as it was beyond the scope of this article. Publication bias exists as non‐published material and conference proceedings were excluded.

Future researchers investigating the role of obesity in RALP outcomes should focus on three key data categories: functional, oncological and peri‐operative. More research is needed on alternative obesity metrics. Obese patients have larger prostates,^16^, ^24^ and glands more than 75 cc are deemed difficult to resect.^30^ Park et al.^18^ have examined a positive relationship between increased WC and larger prostates in prostate cancer patients. This suggests that WC may be a more specific tool for predicting surgical outcomes. This is supported by Sarychev et al.,^9^ who recommend future research focus on central adiposity measurement modalities.

This review highlights a research gap, noting that BMI is the sole anthropometric measure of obesity studied in the RALP population. The distribution of body fat in the abdomen and pelvis complicates RALP, and BMI may not accurately reflect clinically relevant surgical outcomes due to its inability to specify fat distribution. Other studies suggest that measurements like VFA may be superior. Future research should explore other anthropometric tools specific to the target area, such as VFA, WC or WHR, and assess them comprehensively by evaluating functional, oncological and perioperative outcomes.

## AUTHOR CONTRIBUTIONS

Ashley Lee conceived and designed the study, conducted the literature search, performed data extraction and analysis, and drafted and finalised the manuscript. Elizabeth Crostella contributed to data analysis and synthesis and assisted with manuscript preparation. Ann Grand provided oversight during content and manuscript drafting and approved the final version. Matthew Brown originated the research idea, supervised the project, and approved the final manuscript. All authors meet the ICMJE criteria for authorship, have reviewed and approved the final version, and agree to be accountable for all aspects of the work.

## CONFLICT OF INTEREST STATEMENT

The authors declare that they have no conflict of interest.

## Supporting information


**Table S1.** Search strategy.

**Table S2.** Newcastle‐Ottawa Quality Assessment Scale of Cohort Studies.
