# Supplementary material for: Anthropometric assessment of obesity in robotic‐assisted laparoscopic prostatectomy: A systematic review
Source: BJUI Compass. 2026 May 11;7(5):e70206. doi: 10.1002/bco2.70206 (PMC13160930; doi:10.1002/bco2.70206)
Supplement: Supplementary file 1 — Table S1. Search strategy. [file BCO2-7-e70206-s001.docx]

# Supplemental material

Table 1- search strategy

| **Keywords** |  | | |
| --- | --- | --- | --- |
|  | **obesity** | **robotic assisted laparoscopic prostatectomy** | **clinically relevant surgical outcome*** |
|  | **obese** | **robotic prostatectomy** | **blood loss** |
|  | **obesity measurement tool*** | **robotic laparoscopic radical prostatectomy** | **bleeding** |
|  | **obesity metric*** | **rlrp** | **operative time** |
|  | **anthropometric** | **ralp** | **blood transfusion** |
|  | **overweight** |  | **blood products** |
|  | **visceral fat adiposity** |  | **complications** |
|  | **adipos*** |  | **intraoperative complications** |
|  | **visceral adiposity** |  | **postoperative complications** |
|  | **abdominal circumference** |  | **margin status** |
|  | **skin fold thickness** |  | **positive margin** |
|  | **body fat percentage** |  | **biochemical recurrence** |
|  | **waist to hip ratio** |  | **length of stay** |
|  | **waist circumference** |  | **open conversion rates** |
|  | **bmi** |  | **time with catheter** |
|  | **body mass index** |  | **estimated blood loss** |
|  | **dual energy x-ray absorptiometry** |  |  |
|  | **dexa** |  |  |
|  | **bipolar bioelectrical impedance analysis** |  |  |
|  | **bia** |  |  |
|  |  |  |  |
| **MeSH** |  | | |
|  | **Obesity/** | **Robotic Surgical Procedures/ AND (**[**Laparoscopy**](https://aus01.safelinks.protection.outlook.com/?url=https%3A%2F%2Fmeshb.nlm.nih.gov%2Frecord%2Fui%3Fui%3DD010535&data=05%7C02%7C24035886%40student.uwa.edu.au%7C53f7ae51f3cc4af2d28208dd6ce3ad64%7C05894af0cb2846d8871674cdb46e2226%7C0%7C0%7C638786447382935952%7CUnknown%7CTWFpbGZsb3d8eyJFbXB0eU1hcGkiOnRydWUsIlYiOiIwLjAuMDAwMCIsIlAiOiJXaW4zMiIsIkFOIjoiTWFpbCIsIldUIjoyfQ%3D%3D%7C0%7C%7C%7C&sdata=GNnLuH2YBvZi9AEz6ZuhBd6pJ%2BPfGj1o1luaBXaTvaI%3D&reserved=0)**/ OR Prostatectomy/OR Prostate / su OR Prostatic Neoplasms / su)** | **Postoperative Complications/** |
|  | **Adiposity/** |  | **Intraoperative Complications/** |
|  | **Overweight/** |  | **Treatment Outcome/** |
|  | **Waist Circumference/** |  | **Operative Time/** |
|  | **Body Mass Index/** |  | **Length of Stay/** |
|  | **Waist-Hip Ratio/** |  | **Catheters/** |
|  | **Electric Impedance/** |  |  |
|  | **Absorptiometry, Photon/** |  |  |
|  | **Intra-Abdominal Fat/** |  |  |
|  | **Anthropometry/** |  |  |
|  | **Skinfold Thickness/** |  |  |
|  |  |  |  |
| **Embase** |  | | |
|  | **obesity/** | **robot assisted surgery/ AND (laparoscopy/ OR prostatectomy/ OR prostate surgery/)** | **postoperative complication/** |
|  | **waist circumference/** |  | **treatment outcome/** |
|  | **body mass/** |  |  |
|  | **waist hip ratio/** |  |  |
|  | **anthropometry/** |  |  |
|  | **dual energy X ray absorptiometry/** |  |  |
|  |  |  |  |
