# Supplementary material for: Anthropometric assessment of obesity in robotic‐assisted laparoscopic prostatectomy: A systematic review
Source: BJUI Compass. 2026 May 11;7(5):e70206. doi: 10.1002/bco2.70206 (PMC13160930; doi:10.1002/bco2.70206)
Supplement: Supplementary file 2 — Table S2. Newcastle‐Ottawa Quality Assessment Scale of Cohort Studies. [file BCO2-7-e70206-s002.docx]

Supplementary material -2

Table 2 – Newcastle-Ottawa Quality Assessment Scale of Cohort Studies
